# Editorial: creative CRISPR-Cas: RNA-guided functions in defence and beyond

**DOI:** 10.1093/femsml/uqag026

**Published:** 2026-07-11

**Authors:** Wolfgang R Hess, Anita Marchfelder, Lennart Randau

**Affiliations:** Genetics and Experimental Bioinformatics, Faculty of Biology, Freiburg University, 79104 Freiburg, Germany; Molecular Biology and Biotechnology of Prokaryotes, Ulm University, 89069 Ulm, Germany; Prokaryotic RNA Biology, Department of Biology, Philipps-Universität Marburg, 35043 Marburg, Germany

CRISPR-Cas systems are most widely recognized as adaptive immune systems that protect bacteria and archaea from viruses, plasmids, and other mobile genetic elements. Yet, as the field has advanced, it has become increasingly clear that CRISPR-Cas biology cannot be contained within a simple defence paradigm. CRISPR-Cas components shape genome evolution, influence DNA repair, contribute to gene regulation, affect host physiology, and provide increasingly versatile tools for diverse applications in organisms across the tree of life. This special issue of *microLife* brings together a collection of research articles and reviews that highlight this breadth under the theme “CRISPR-Cas functions in defence and beyond”. Together, the contributions show how mechanistic studies, bioinformatic approaches, comparative genomics, metagenomics, single-cell imaging, and genetic tool development are expanding our view of CRISPR-Cas systems from immune modules to integrated components of microbial life.

Several studies in this issue examine how CRISPR-Cas defence can be observed, detected, and interpreted across scales. Rust and Randau (2025) introduce SMARTIS, a smartphone-based approach for real-time imaging of bacterial colony growth during Type IV-A1 CRISPR-Cas activity. Their work reveals that plasmid targeting can restrict colony growth without the DNA degradation typical of some other CRISPR-Cas systems. This study emphasizes that CRISPR-Cas activity is not always a uniform population-level phenotype but can produce heterogeneity that becomes visible only through time-resolved observation.

At the level of microbial communities, Talibli and Voß (2025) present the Metagenomic CRISPR Array Analysis Tool, an assembly-free, graph-based method for identifying CRISPR arrays directly in metagenomic datasets. By avoiding reliance on genome assembly, this approach improves access to CRISPR diversity in complex communities and opens new opportunities for tracking spacer dynamics, microbial host-virus interactions, and CRISPR array evolution in environmental datasets. Complementing this computational advance, Fehrenbach et al. (2026) explore the complexity of multiple CRISPR arrays in genomes with single or co-occurring CRISPR-Cas systems. Their analyses show that array number, position, repeat diversity, and spacer acquisition patterns vary across system types, suggesting that multiple arrays may distribute immune memory in ways that reflect both system architecture and evolutionary history. Mitrofanov et al. (2026) further deepen this evolutionary perspective through a large-scale analysis of repeat mutations across CRISPR arrays. Their finding that repeat mutation patterns are subtype-specific and linked to spacer dynamics underscores that even the repeat sequences forming the structural backbone of CRISPR arrays carry an evolutionary signal.

A second group of contributions focuses on RNA processing, RNA control and the regulatory potential of CRISPR-Cas-associated RNAs. Feussner et al. (2025) examine how type II-C CRISPR-Cas systems suppress the production of extraneous CRISPR RNAs. By identifying distinct strategies, including transcriptional termination, guide sequestration, and repeat mutations, their study highlights the importance of controlling CRISPR RNA output to maintain effective and specific immune function. Bilger et al. (2025) investigate CRISPR RNA maturation in the cyanobacterium *Synechocystis* sp. PCC 6803 and identify RNase J as an additional enzyme acting together with RNase E in the maturation of type III-Bv CRISPR RNAs. These findings expand the set of host factors known to contribute to CRISPR RNA biogenesis and illustrate how CRISPR function depends on broader RNA-processing networks.

Ziemann et al. (2025) extend this theme into the diverse world of CRISPR-associated transposons. Their analysis of type V-K CAST systems identifies a new subgroup, V-K_V2, distinguished by alternative tracrRNAs, distinct regulatory features and signatures of horizontal gene transfer. This work refines our understanding of RNA-guided transposition and provides a framework for exploring how CAST systems diversify in bacterial genomes. Moreover, the integration of type V-K _V2 CAST tracr RNAs was taken as an opportunity to release a new version 2.0 of the popular CRISPRtracrRNA algorithm to facilitate the efficient genome-wide identification of tracrRNA sequences. Arifah et al. (2025) show that a short tracrRNA can be cytotoxic in *Lacticaseibacillus paracasei* independently of Cas9, linking toxicity to interactions between the tracrRNA and host RNAs. Their study provides an important reminder that CRISPR-associated RNAs can have physiological effects beyond their canonical roles in guiding Cas nucleases. In the same spirit, Pytlik et al. (2026) extend CRISPR-associated regulation to *Neisseria meningitidis*, where a CRISPR-Cas-associated scaRNA together with Nme1Cas9 dampens expression of the *efeUOB* operon, fine-tuning iron uptake and oxidative stress responses. These studies collectively reveal that CRISPR RNAs and CRISPR-associated RNAs are not merely immune intermediates but can also intersect with host regulatory circuits and shape microbial physiology.

The archaeal contributions in this issue illustrate especially well how CRISPR-Cas systems connect immunity, mobile genetic elements, genome maintenance, and microbial evolution. Di Cianni et al. (2025) investigate the consequences of deleting proviruses from *Haloferax volcanii*. Their work shows that proviruses are not essential under laboratory conditions, but that their removal affects motility, stress resistance, cell morphology, and CRISPR RNA expression. The detection of circularized viral genomes, viral particles, and viral proteins further supports the view that proviruses can remain active components of archaeal genome biology. Naki and Gophna 2025 then examine provirus-encoded CRISPR-Cas systems in halophilic archaea and propose that some proviral systems may rely on an host-encoded adaptation machinery, a concept they describe as adaptive outsourcing. Such findings blur the boundary between host defence and mobile element biology, i.e. CRISPR-Cas systems can defend against mobile elements, but they can also be encoded, shaped, and repurposed by them.

Choudhary et al. (2026) add another layer by showing that CRISPR-Cas targeting in *H. volcanii* can promote within-species gene exchange by triggering homologous recombination. Rather than simply preventing DNA entry, CRISPR targeting can bias gene transfer outcomes in favour of compatible partners and may reduce exchange between species. This work highlights a role for CRISPR-Cas systems in structuring gene flow and potentially contributing to microbial speciation. Sailer et al. (2025) use CRISPR-Cas-induced self-targeting as a genetic entry point to identify key factors involved in archaeal microhomology-mediated end joining. Their study connects CRISPR self-targeting lesions to DNA repair and identifies proteins including Mre11, Rad50, Fen1, PolB1, LigA, LigN, Cas1, and Hel308a as contributors to repair outcomes. Rentz et al. (2025) provide functional insight into Solo-Cas4 in *Methanosarcina mazei* Gö1. Their biochemical and physiological analyses show that this standalone Cas4 protein has metal-dependent nuclease activity, forms higher-order complexes and affects growth when overexpressed, suggesting that solo Cas proteins may have functions that remain underappreciated outside canonical CRISPR-Cas loci.

Several articles also demonstrate how CRISPR-Cas biology is becoming inseparable from the development of new experimental systems. de Pierpont et al. (2025) expand the genetic toolbox of the obligate predatory bacterium *Bdellovibrio bacteriovorus* through inducible gene expression and CRISPR interference. Their work enables controlled knockdown of genes in an organism that has historically been difficult to manipulate genetically, and they use these tools to interrogate functions including cell shape and division. In archaea, Schrage et al. (2026) introduce a new expression system for single-molecule fluorescence imaging in *H. volcanii* WR806. By visualizing Cas1 dynamics during UV-induced DNA damage, they provide evidence that Cas1 behaviour changes during DNA damage response.

Finally, the review by Mitousis et al. (2025) places CRISPR-Cas systems in *Actinomycetes* into a broader evolutionary and biotechnological context. *Actinomycetes* are renowned for their ecological importance and natural product biosynthesis, yet their CRISPR-Cas systems remain comparatively underexplored. The review highlights both the diversity of CRISPR-Cas loci in this group and the limited number of experimentally characterized examples. It also points to unusual maturation pathways, possible lifecycle-dependent activity and the promise of endogenous CRISPR-Cas systems for genetic manipulation. This synthesis reinforces a central message of the special issue, highlighting that many of the most informative mechanistic variations of CRISPR-Cas system activity remain to be discovered outside the realm of traditional model organisms.

Taken together, the manuscripts in this special issue show that CRISPR-Cas systems are best understood as dynamic biological modules embedded in microbial genomes, physiologies, and communities. Their roles in defence remain fundamental, but there is so much more to discover: CRISPR-Cas systems record encounters with mobile genetic elements, influence the flow of genes within and between populations, rely on host RNA-processing pathways, are deeply embedded in cellular regulatory networks, contribute to DNA repair phenotypes and inspire new tools for studying widely different organisms. Many articles of the special issue “CRISPR-Cas functions in defence and beyond” therefore capture not a departure from immunity, but an expansion of it, as the immune function of CRISPR-Cas is deeply connected to the wider biology of the cell.

The special issue editors are grateful to the DFG for funding the priority programme SPP 2141, to all authors who contributed their work to this collection and to the reviewers whose careful assessments helped ensure the quality of the published manuscripts. We hope that this issue will encourage further exploration of CRISPR-Cas systems across the entire spectrum of microbial life, from well-established model organisms to environmentally, medically and evolutionarily important microbes that are only beginning to reveal their CRISPR-Cas biology.
